# Improvement of Sourdough and Bread Qualities by Fermented Water of Asian Pears and Assam Tea Leaves with Co-Cultures of *Lactiplantibacillus plantarum* and *Saccharomyces cerevisiae*

**DOI:** 10.3390/foods11142071

**Published:** 2022-07-12

**Authors:** Ramita Supasil, Uthaiwan Suttisansanee, Chalat Santivarangkna, Nattapol Tangsuphoom, Chanakan Khemthong, Chaowanee Chupeerach, Nattira On-nom

**Affiliations:** Food and Nutrition Academic and Research Cluster, Institute of Nutrition, Mahidol University, Salaya, Phuttamonthon, Nakhon Pathom 73170, Thailand; ramita.sup1@gmail.com (R.S.); uthaiwan.sut@mahidol.ac.th (U.S.); chalat.san@mahidol.ac.th (C.S.); nattapol.tng@mahidol.ac.th (N.T.); chanakan.khe@mahidol.ac.th (C.K.); chaowanee.chu@mahidol.ac.th (C.C.)

**Keywords:** *Pyrus pyrifolia*, *Camellia sinensis* var. *assamica*, fermentation, nutritive composition, physical properties, chemical properties, antioxidant activities, sensory properties, shelf-life

## Abstract

Qualities of sourdough and sourdough bread using fermented water from Asian pears and Assam tea leaves with *Lactiplantibacillus plantarum* 299v and *Saccharomyces cerevisiae* TISTR 5059 as starter cultures were evaluated. Changes in the growth of lactic acid bacteria and yeast, pH, sourdough height, total phenolic contents (TPCs) and antioxidant activities detected by ORAC, FRAP and DPPH radical scavenging assays were monitored during sourdough production. Mature sourdough was achieved within 4 h after 18 h retard fermentation and used for bread production. The bread was then analyzed to determine chemical and physical properties, nutritional compositions, TPCs, antioxidant activities and sensory properties as well as shelf-life stability. Results showed that fermented water significantly promoted the growth of yeast and increased TPCs and antioxidant activities of sourdough. Compared to common sourdough bread, fermented water sourdough bread resulted in 10% lower sugar and 12% higher dietary fiber with improved consumer acceptability; TPCs and antioxidant activities also increased by 2–3 times. The fermented water sourdough bread maintained microbial quality within the standard range, with adequate TPCs after storage at room temperature for 7 days. Fermented water from Asian pears and Assam tea leaves with *L. plantarum* 299v and *S. cerevisiae* TISTR 5059 as starter cultures improved dough fermentation and bread quality.

## 1. Introduction

Nowadays, consumers prefer to eat healthy food [1]. Sourdough bread offers health benefits with enhanced absorbable nutrients and vitamins [2,3] and reduced glycemic index value compared to common white bread [4]. Cereal flour and water as the main ingredients of traditional sourdough are spontaneously fermented, enhancing numbers of wild yeast and lactic acid bacteria [5]. The combination of culture in the fermentation provides complex growth patterns that can improve the functional and organoleptic properties of food [6]. *Saccharomyces cerevisiae* is a robust yeast that has a high fermentation efficiency [7]. It acts as a leavening agent in bread making [5]. *Lactiplantibacillus plantarum* has been recognized for its probiotic characteristics. It has been used widely in food fermentation processes of different types of foods [8]. In bread making, lactic acid bacteria contribute mainly to acidification and the production of flavor and other metabolic compounds of bread [5]. The study of Hu et al., (2022) reported that the bread fermented by a combination strains of *S. cerevisiae* and *L. plantarum* had 15.2% higher specific volume, softer bread crumb, more vigorous taste than the bread fermented by *S. cerevisiae* only [9]. In addition, functional ingredients may also be introduced in the early fermentation steps to further improve the functional properties of the final product [10]. The several studies showed that sourdough was developed using pacific white shrimp (*Litopenaeus vannamei*) protein hydrolysates and (−)-epigallocatechin gallate [11], pear and naval orange [12]. Sources of these functional ingredients as nutrients or bioactive compound-rich fruits and vegetables support bacterial growth and provide particular taste/texture to increase the health benefits of sourdough bread.

The abundant nutrients in Asian pears and Assam tea leaves can be used to support the growth of microorganisms [13,14]. Asian pears (*Pyrus pyrifolia*) are juicy fruits with favorable aroma and sweetness that enhance the sensory acceptability of developed products [15]. Pears are an excellent source of dietary fiber and a good source of ascorbic acid [16]. Moreover, it contains a high number of bioactive compounds including total phenolic compounds and total flavonoid compounds which significantly correlated with antioxidant activities [17]. Assam tea (*Camellia sinensis* var. *assamica*) leaves also possess a unique aroma and taste. It contains a variety of biologically active compounds, such as phenolics, minerals, vitamins and dietary fiber, and have several health benefits including antimicrobial and antioxidant activities [13,18,19]. As far as we know, a study of fermented water from Asian pears and Assam tea leaves using *Lactiplantibacillus plantarum* 299v and *Saccharomyces cerevisiae* TISTR 5059 as starter on sourdough fermentation and bread quality has not been reported. Recent investigations by our group indicated that fermented water from Asian pears and Assam tea leaves using *Lactiplantibacillus plantarum* 299v and *Saccharomyces cerevisiae* TISTR 5059 as starter cultures significantly promoted the growth of yeast and lactic acid bacteria, optimized acidity and improved total phenolic contents (TPCs) and antioxidant activities [20]. Previous findings suggested the potential of fermented water from Asian pears and Assam tea leaves as a functional ingredient to prepare sourdough and improve bread quality.

Hence, the aim of this work was to develop sourdough and bread with improved qualities using fermented water from Asian pears and Assam tea leaves using *L. plantarum* 299v and *S. cerevisiae* TISTR 5059 as starter cultures. Changes in microbiological quantification, chemical properties, TPCs and antioxidant activities were monitored during sourdough fermentation. Mature sourdough was used for breadmaking. Chemical and physical properties, nutritional compositions, TPCs, antioxidant activities and sensory evaluation of sourdough bread were investigated. Shelf-life stability was also determined.

## 2. Materials and Methods

### 2.1. Preparation of Fermented Water

Asian pears were purchased from Salaya Market, Phutthamonthon District, Nakhon Pathom Province, Thailand and steamed Assam tea leaves were received from Wiang Pa Pao District, Chiang Rai Province, Thailand in December 2020. For quality control of raw material, nutritional analysis was performed according to the protocols indicated in Section 2.7. The chemical composition of Asian pears was 84.73% moisture, 0.45% protein, 0.11% total fat and 14.11% total carbohydrates (comprising 5.18% glucose, 5.11% fructose, 0.75% sucrose and 2.80% dietary fiber), while steamed Assam tea leaves were composed of 77.32% moisture, 5.32% protein, 0.48% total fat and 15.44% total carbohydrates (comprising 0.75% sucrose and 8.03% dietary fiber). Fermented water was prepared following the method of Supasil et al., (2021) [20]. Briefly, Asian pears (50 g) and steamed Assam tea leaves (50 g) with suspensions of *L. plantarum* 299v (6 mL, Bio-Life Sciences Corp., Mississauga, Canada) and *S. cerevisiae* TISTR 5059 (6 mL, TISTR, Bangkok, Thailand) were mixed with distilled water (488 mL) and fermented in a sterile glass jar covered with a lid at 30 °C for 24 h. Then, solid remnants of Asian pears and Assam tea leaves were filtered using a sterile colander. The solution part of the fermented water was used in fermented water sourdough and fermented water sourdough bread development. In addition, a suspension of *L. plantarum* 299v and *S. cerevisiae* TISTR 5059 in sterile deionized water was prepared as control water and used in common sourdough and common sourdough bread development. Initial viable cell count of *L. plantarum* at 8.56 ± 0.16 Log CFU/mL and *S. cerevisiae* at 7.23 ± 0.05 Log CFU/mL in the suspension of selective cultures were maintained in both control and fermented water (*p* ≥ 0.05).

### 2.2. Preparation of Sourdough

Fermented water sourdough was prepared by mixing unbleached wheat flour (200 g, Bread Flour, Thai Flour Mill Industry Co., Ltd., Samut Prakan, Thailand) with fermented water from Section 2.1 (200 g) in a sterile glass jar (5.2 inches diameter and 6.5 inches height) covered by a cheesecloth. The mixture was fermented at 30 °C for 1 h, followed by retard fermentation in a refrigerator (7 ± 2 °C) for 18 h. The final fermentation was performed at 30 °C to produce mature sourdough with the following characteristics: pH 3.5–4.3, lactic acid bacteria at least 8 Log Colony Forming Unit (CFU)/g and yeast at least 6 Log CFU/g according to Hammes et al., (2005) [21] and Mohd Roby et al., (2020) [22]. Development of bubbles, sweet smelling odor and double volume was observed. Common sourdough was prepared using a similar procedure to the fermented water sourdough but fermented water was replaced by control water from Section 2.1. The sourdough samples were observed for their height, collected for evaluation of microbiological and chemical properties at initiation and then hourly after retard fermentation until gaining the mature sourdough. Moreover, TPCs and antioxidant activities at the start and end of the fermentation were also analyzed.

### 2.3. Preparation of Bread

The fermented water sourdough bread and common sourdough bread were prepared using the mature fermented water sourdough and mature common sourdough from Section 2.2 as a leavening agent. The recipe of sourdough bread, modified from Mohd Roby et al., (2020) [22], consisted of unbleached wheat flour (100%, Bread Flour, Thai Flour Mill Industry Co., Ltd., Samut Prakan, Thailand), distilled water (44%), sugar (15%, Lin Caster Sugar, Thai Roong Ruang Sugar Group, Bangkok, Thailand), salt (2.5%, Prung Thip Iodized Table Salt, Thai Refined Salt Co., Ltd., Bangkok, Thailand), butter (5%, Allowrie Pure Creamy Unsalted Butter, KCG Corporation Co., Ltd., Bangkok, Thailand) and mature sourdough (44%). All ingredients, with the exception of butter, were mixed and kneaded for 5 min by a dough kneading machine (model Bear HMJ-A50E2, Bear Electric Appliance Co., Ltd., Shunde, Fosahn, China). The butter was then added to the mixture, kneaded for another 25 min, and allowed to proof for 4 h at 30 °C. The dough was then portioned (approximated 175 g), rested at room temperature (28 ± 2 °C) and 75 ± 5% relative humidity (RH) for 20 min, rolled for degassing and shaped into a roll. Three rolls were placed into a loaf pan (8.5 × 14.5 × 8.5 cm) before overnight leavening in a refrigerator (7 ± 2 °C) and final proofing at 30 °C for 6 h. The doughs were then baked at 180 °C for 30 min using an electric oven (model Tecno+, The Signature Brand Co., Ltd., Bangkok, Thailand) and cooled at room temperature (28 ± 2 °C and 75 ± 5% RH) for 1 h before packing in polypropylene plastic bags (Aro Commercial Co., Ltd., Bangkok, Thailand) with a proper heat seal for further analysis. The bread samples were analyzed for chemical and physical properties, nutritional compositions, TPCs, antioxidant activities and sensory evaluation.

### 2.4. Determination of Microbiological Quality

Sourdough samples (10 g fresh weight (FW)) were suspended in 90 mL of 0.85% (*w/v*) sodium chloride solution and enumerated for *L. plantarum* and *S. cerevisiae* by spread plate technique [23]. *L. plantarum* grew on De Man Rogosa Sharpe (MRS) (Difco™ & BBL™, BD Diagnostic, Sparks, MD, USA) agar (Hardy Diagnostics, Santa Maria, CA, USA) supplemented with 4 mg/L ciprofloxacin (Siam Pharmaceutical Co., Ltd., Bangkok, Thailand), incubated at 37 °C for 48 h [24]. *S. cerevisiae* grew on Yeast Peptone Dextrose (YPD) agar supplemented with 100 mg/L chloramphenicol (T.P. Drug Laboratories (1969) Co., Ltd., Bangkok, Thailand), incubated at 30 °C for 48 h [25]. Results were calculated and reported as Log CFU/g.

Microbiological analysis of the bread samples was performed according to the standard protocols of the Bacteriological Analytical Manual (BAM) [26]. Bread samples were blended using a blender (model HR2115, Philips (Thailand) Ltd., Bangkok, Thailand). Ten grams of blended sample were suspended in 90 mL of 0.1% (*v/v*) peptone water, plated on Dichloran-Glycerol 18 (DG18) agar and incubated at 25 °C for 5 days in the dark for yeast and mold evaluation. Total aerobic bacteria were evaluated following a spread plate technique [23] and BAM [26]. Briefly, the blended samples were suspended in 0.85% (*w/v*) sodium chloride solution, poured onto plate count agar and incubated at 37 °C for 48 h. Results were calculated and reported as Log CFU/g.

### 2.5. Determination of Chemical and Physical Quality

For pH measurement, the sourdough or blended bread samples (10 g) were mixed with 90 mL of 0.85% (*w/v*) sodium chloride solution and measured for pH using a calibrated pH meter (Ohaus Corporation, Morris Country, NJ, USA) [25]. Specific volume was calculated using loaf volume divided by loaf weight. Loaf weight was measured by an electronic weighing balance (Mettler Toledo, Toronto, Canada), while loaf volume was determined using the black sesame seeds replacement method [11,27]. The blended bread samples were measured for water activity by a water activity measurement instrument (model ms1-1 M, Novasina, Lachen, Switzerland). Texture of the bread samples including hardness, chewiness, springiness and cohesiveness was measured as described by Mohd Roby et al., (2020) [22] and the American Association of Cereal Chemists (AACC) (2001) [28] with some modifications as follows. Three slices of bread including the middle slice and one on either side were used for texture profile analysis (TPA) using a Texture Analyzer (TA.XT.plus^®^, Stable Micro System, Surrey, UK) equipped with an aluminum 36-mm cylindrical probe with the following parameters: 1.0 mm/s pre-test speed and 0.5 mm/s test speed, 10.0 mm/s post-test speed, 5 mm distance, 5.0 s time and 5.0 g trigger force.

### 2.6. Determination of Total Phenolic Contents and Antioxidant Activities

The sourdough samples were collected to evaluate TPCs and antioxidant activities at the start and end of the fermentation, while the bread samples were collected every day for 7 days. The collected samples were freeze-dried using a −50 °C and 0.086 mbar freeze dryer (model Lyovac GT2, GEA^®^ Lyophil GmbH, Nordrhein-Westfalen, Germany) for 72 h and ground using a grinder (model MR-1268, MARA, Nonthaburi, Thailand) into fine powder. Extraction of all samples followed the method of Sripum et al., (2017) [29] with slight modifications as follows. The powdered sample (1 g) was dissolved in 40% (*v/v*) aqueous ethanol (20 mL) and incubated at 50 °C using a WNE45 water bath shaker (Memmert GmBh, Eagle, WI, USA) for 2 h. The supernatant was collected by centrifugation at 3800× *g* using a Hettich^®^ ROTINA 38R centrifuge (Andreas Hettich GmbH, Tuttlingen, Germany) for 10 min and filtered through a 0.45 µM polyether sulfone membrane syringe filter. The filtrate was kept at −20 °C until analysis.

TPCs and antioxidant activities were assessed following Sripum et al., (2017) [29] with no modifications. Briefly, Folin-Ciocalteu phenol was used as the reagent, while gallic acid (0 to 200 µg/mL) was used as a standard for the determination of TPCs. A Synergy^TM^ HT 96-well UV/visible microplate reader with Gen 5 data analysis software (BioTek Instruments, Inc., Winooski, VT, USA) was used to detect TPCs at 765 nm, and the results were reported as mg gallic acid equivalent (GAE)/g dry weight (DW). Antioxidant activities were analyzed for oxygen radical absorbance capacity (ORAC), ferric ion reducing antioxidant power (FRAP) and 2,2-diphenyl-1-picrylhydrazyl (DPPH) radical scavenging assays. Fluorescein, FRAP reagent and DPPH in 95% (*v/v*) aqueous ethanol were employed as reagents for the ORAC, FRAP and DPPH radical scavenging assays, respectively. Antioxidant activities were monitored at an excitation wavelength (λ_ex_) of 485 nm and an emission wavelength (λ_em_) of 528 nm for ORAC assay, 600 nm for FRAP assay and 520 nm for DPPH radical scavenging assay. Trolox was used as a standard in all antioxidant assays and results were reported as µmol Trolox equivalent (TE)/g DW. All chemicals and reagents were sourced from Sigma-Aldrich (St. Louis, MO, USA).

### 2.7. Determination of Nutritional Quality

Determination of nutritional compositions (moisture, fat, protein, carbohydrate, energy, dietary fiber, sugar and ash) was conducted at the Institute of Nutrition, Mahidol University (Nakhon Pathom, Thailand) using the international standard for laboratory quality systems with ISO/IEC 17025:2005 and the standard protocols of the Association of Official Analytical Chemists (AOAC) [30]. Moisture content was evaluated by drying the fresh samples in a hot-air oven (Memmert model UNE 500, Eagle, WI, USA) at 100 °C until constant weight (AOAC 930.04, 934.01). Total fat content was determined by acidic digestion and extracted with petroleum ether using a Soxtec System (Tecator model 1043, Hoganas, Sweden) (AOAC 948.15, 945.16). Protein content was analyzed by the Kjeldahl method utilizing digestion and distillation units (Buchi model K-435 and B-324, Flawil, Switzerland, respectively), and then calculated using a conversion factor of 6.25 (AOAC 992.23). Ash content was analyzed by incineration in a muffle furnace (Carbolite model CWF 1100, Hope, UK) at 550 °C (AOAC 930.30, 945.46). Total carbohydrate was calculated by the subtraction of moisture, fat, protein and ash contents from 100. Energy value was attained from the integration of total energy from carbohydrate, protein and fat as 4, 4 and 9 kcal/g samples, respectively. Total dietary fiber was evaluated by the enzyme gravimetric method (AOAC 991.43). Total sugar was determined using a protocol previously reported by Wannasaksri et al., (2021) [31] as a liquid chromatographic method utilizing ultra-fast liquid chromatography (UFLC from Shimadzu Corporation, Kyoto, Japan) with a detector (Alltech 800 evaporative light scattering detector from BÜCHI Corporation, New Castle, DE, USA) and column (5 µm, 250 mm × 4.6 mm Shodex Asahi Pak NH2P-504E from Shodex Group, Kanagawa, Japan).

### 2.8. Sensory Evaluation

Fermented water sourdough bread and common sourdough bread were evaluated for sensory properties by 10 trained panelists (4 male and 6 females, range: 18 to 60 years old, nonsmokers), who were familiar with sourdough bread. A 9-point hedonic scale rating 1 for dislike extremely, 5 for neither like nor dislike and 9 for like extremely was utilized to evaluate consumer attributes including appearance, color, taste, aroma, texture, sourness and overall acceptability [32]. The panelists were allowed to drink water for mouth cleansing between sample testing.

### 2.9. Shelf-Life Stability

The bread samples were packed in clear polypropylene plastic bags with a proper heat seal and stored at room temperature (28 ± 2 °C and 75 ± 5% RH). Samples were randomly selected to evaluate microbiological, TPCs and antioxidant activities at 0, 1, 3, 5 and 7 days. For microbiological properties, total aerobic bacteria, as well as yeast and mold, were enumerated by the pour plate technique, as described in Section 2.4, while TPCs and antioxidant activities were analyzed using the protocols listed in Section 2.6.

### 2.10. Statistical Analysis

All experiments were performed in triplicate using three independent batches of sourdough and bread, with data presented as mean ± standard deviation (SD) of each quality value and subjected to univariate data analysis using IBM SPSS Statistics for Windows version 26.0, IBM Corp., Armonk, New York, USA software. Mean differences of *p* < 0.05 were determined by one-way analysis of variance (ANOVA), followed by Duncan’s multiple comparison test for more than two data sets or Student’s unpaired *t*-test for two data sets.

## 3. Results and Discussion

### 3.1. Effect of Fermented Water and Fermentation Time on Sourdough Quality

#### 3.1.1. Growth of *L. plantarum* and *S. cerevisiae*

**Figure 1:**
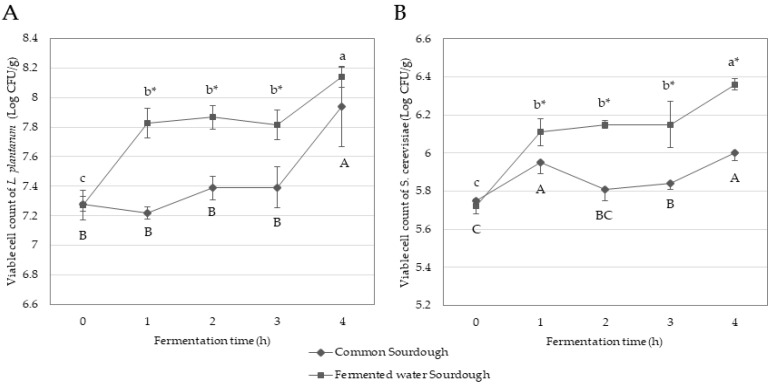
Growth of (**A**) *L. plantarum* and (**B**) *S. cerevisiae* in common sourdough and fermented water sourdough throughout fermentation. Results were expressed as mean values of triplicate determinations (*n* = 3). Different uppercase and lowercase letters denote significantly different viable cell count of microorganisms in common sourdough and fermented water sourdough, respectively, at different fermentation times using one-way ANOVA, followed by Duncan’s multiple comparison test at *p* < 0.05, while * denotes significantly different viable cell count of microorganisms between common sourdough and fermented water sourdough detected at the same fermentation time using Student’s unpaired *t*-test at *p* < 0.05.

The initial viable cell count of *L. plantarum* and *S. cerevisiae* in the fermented water sourdough was 7.27 ± 0.10 and 5.72 ± 0.04 Log CFU/g, respectively, which were not significantly different (*p* ≥ 0.05) from the common sourdough (Figure 1 and Supplementary Table S1). It seemed that the viable cell counts of *L. plantarum* and *S. cerevisiae* increased rapidly in the fermented water sourdough than the common sourdough in the beginning stage (1 h) of fermentation. This could be due to the available nutrients in the fermented water or unbleached wheat flour as carbon or nitrogen sources that enhanced bacterial growth [20]. In the later stage (2–3 h) of fermentation, less rapid growth was observed. This might be because of pH which is an imporatant controlling factor for the survival and growth of the microorganisms [33]. The growth rate of *L. plantarum* and *S. cerevisiae* increased again in the fourth h of fermentation and reached a maximum number of viable cell count. Both common sourdough and fermented water sourdough achieved the criteria of mature sourdough pH between 3.5 to 4.3, lactic acid bacteria at least 8 Log CFU/g and yeast at least 6 Log CFU/g [21,22] within 4 h after 18 h retard fermentation. Viable cell count of *L. plantarum* reached a maximum of 7.94 ± 0.27 Log CFU/g in common sourdough and 8.14 ± 0.07 Log CFU/g in fermented water sourdough. A similar growth trend with lower viable cell count of *S. cerevisiae* was observed. Growth rate gradually increased and reached a maximum at 6.00 ± 0.04 Log CFU/g in common sourdough and 6.36 ± 0.03 Log CFU/g in fermented water sourdough. Similar results were reported by Minervini et al., (2016). They found that dough containing macerated pears exhibited 8 Log CFU/g of lactic acid bacteria and 6 Log CFU/g of yeast after 8 h fermentation at 30 °C [34], while Unban et al., (2019) reported that lactic acid bacteria detected in fermented Assam tea leaves (Cha-miang) ranged of 6–8 Log CFU/g [35], with yeast and mold ranging 6–10 Log CFU/g [36]. During fermentation, the fermented water sourdough exhibited significantly higher viable cell count of *L. plantarum* and *S. cerevisiae* than common sourdough. Catechin, a predominant polyphenol in Assam tea leaves, promoted the growth of *L. plantarum* [37]. López de Felipe et al., (2010) explained that absorption of catechin through the lactic acid bacterial membrane altered the function of proteins associated with glucose transport, resulting in increased glucose consumption and higher growth of *L. plantarum* [38]. Nutrients in the fermented water including glucose and fructose enhanced *S. cerevisiae* growth [39]. Another supportive study indicated that *L. plantarum* and *S. cerevisiae* stimulated growth of each other only in the presence of fructose, glucose and lactose as carbon sources but not with galactose, maltose, sucrose and starch [34].

#### 3.1.2. pH and Height

**Figure 2:**
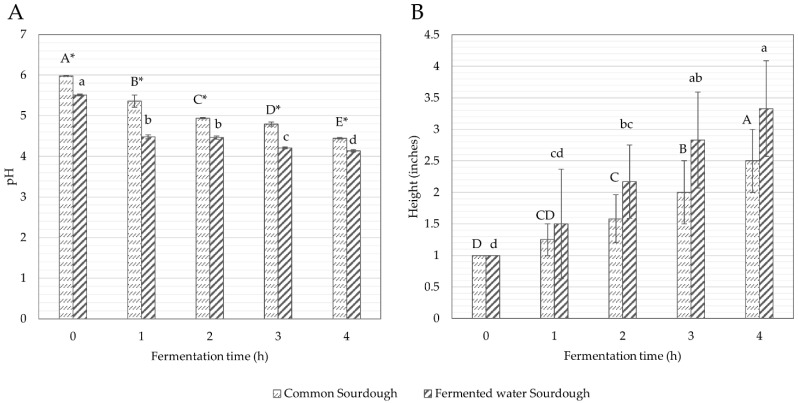
A trend in (**A**) pH and (**B**) height of common sourdough and fermented water sourdough over fermentation time periods. Results were expressed as mean values of triplicate determinations (*n* = 3). Different uppercase and lowercase letters denote significantly different values (pH or height) at *p* < 0.05 in common sourdough and fermented water sourdough, respectively, fermented at different time periods using one-way ANOVA, followed by Duncan’s multiple comparison test, while * denotes significantly different values (pH or height) at *p* < 0.05 between common sourdough and fermented water sourdough fermented at the same time period using Student’s unpaired *t*-test.

The initial pH of fermented water sourdough was 5.51 ± 0.02, and significantly (*p* < 0.05) lower than common sourdough (5.98 ± 0.01) (Figure 2A and Supplementary Table S2). Throughout fermentation, the pH of both common sourdough and fermented water sourdough was significantly (*p* < 0.05) reduced to 4.45 ± 0.02 and 4.14 ± 0.03, respectively, within 4 h due to acid production of *L. plantarum* and *S. cerevisiae*. This result was supported by Jin et al., (2019), who observed that lactic acid was the main organic acid produced (up to 6.12 g/L) in a mixed 24 h fermentation of mango slurry using *L. plantarum* and *S. cerevisiae* DV10 [40]. Similar results were reported by Duan et al., (2011). They revealed that the increased growth rate of *L. plantarum* was influenced by shrimp waste peptides that elevated the rate of lactic acid production, resulting in lower pH [41], while persistent acids from fermented water including lactic acid, butyric acid, caffeic acid and sinapic acid were mainly found in fermented Assam tea leaves [42], with chlorogenic acid, ascorbic acid and sinapic acid found in Asian pears [16,43].

Likewise, initial heights of common sourdough and fermented water sourdough were insignificantly different (Figure 2B). Similar to microorganism growth, sourdough height increased during fermentation, reaching a maximum within 4 h after the 18 h retard fermentation (2.50 ± 0.50 inches in common sourdough and 3.33 ± 0.76 inches in fermented water sourdough). Bubbles and a 2–3 times increment in volume were observed in mature sourdough. The gas produced by *L. plantarum* and *S. cerevisiae* during fermentation resulted in doubling the volume of dough. The results were explained by Winters et al., (2019). They found that the combination of *L. plantarum* and *S. cerevisiae* had an increase in gas produced compared to the yeast alone [44].

#### 3.1.3. Total Phenolic Contents and Antioxidant Activities

At the start of the fermentation, fermented water sourdough had significantly higher (*p* < 0.05) TPCs and antioxidant activities detected by ORAC, FRAP and DPPH radical scavenging assays than common sourdough (Table 1), and also exhibited significantly (*p* < 0.05) higher TPCs and antioxidant activities than common sourdough throughout the fermentation. At the end of the fermentation (4 h after the 18 h retard fermentation), TPCs and antioxidant activities of fermented water sourdough were 2–7 times higher than common sourdough due to the remaining TPCs and antioxidant activities in the fermented water [20]. Moreover, a significantly lower pH in fermented water sourdough than in common sourdough supported bioactive compound stabilization. Złotek et al., (2019) reported that polyphenols were auto-oxidized with increased pH [45].

Moreover, TPCs and antioxidant activities of both sourdough types tended to increase throughout the fermentation (Table 1). Fermentation broke down the structure of unbleached wheat flour or fermented water [46,47], related to significant (*p* < 0.05) growth of *L. plantarum* in both types of sourdough during fermentation, resulting in the activation of complex polyphenol hydrolyzing enzymes that produced simpler and active polyphenols [48]. Katina et al., (2007) reported that amylase, proteases and xylanases derived from microbes and grains during sourdough fermentation released phenolics [49], while Złotek et al., (2019) reported that *L. plantarum* 299v enrichment significantly (*p* < 0.05) improved TPCs in legume sprout preparation through de novo synthesis induction [45]. The results showed that fermentation significantly increases TPCs in both fermented sourdough and common sourdough. Corresponded to increased TPCs, the antioxidant activities of fermented sourdough and common sourdough measured with ORAC and DPPH radical scavenging assay were greatly increased after fermentation. Since ORAC assay is a method to measure antioxidants with hydrogen atom transfer (HAT) mechanism and DPPH radical scavenging assay was for antioxidants with both HAT and single electron transfer (SET) mechanisms, these results suggested that increased phenolics after fermentation might act as antioxidants with HAT mechanism rather than SET mechanism. However, the FRAP activities were slightly reduce in fermented water sourdough after fermentation. Since FRAP assay is for antioxidants following SET mechanism, this result suggested the potential degradation of antioxidants with SET mechanism. A similar result was report by Kinga et al., (2021). They found that after fermentation of natural fruit meads the antioxidant activity measured by FRAP assay (SET mechanism) was reduced by 18%, while those by ABTS^+^ assay increased by 14% (both HAT and SET mechanisms) [50].

### 3.2. Effect of Fermented Water on Bread Quality

#### 3.2.1. Chemical and Physical Quality

Water activity, pH, specific volume and texture of bread are shown in Table 2. No significant (*p* ≥ 0.05) differences in water activity in all bread samples (0.82 ± 0.00) were observed but fermented water sourdough bread showed a greater pH decrease than the control bread obtained from common sourdough (*p* < 0.05). A similar result was reported by Mohd Roby et al., (2020). They found that encapsulated kombucha sourdough bread had a significantly (*p* < 0.05) lower pH than liquid traditional sourdough bread [22], while Karimi et al., (2020) observed a significantly (*p* < 0.05) lower pH in sourdough bread containing (−)-epigallocatechin gallate and pacific white shrimp (*Litopenaeus vannamei*) protein hydrolysates than common sourdough bread (without added ingredients) [11]. A significantly (*p* < 0.05) lower pH of fermented water sourdough bread than common sourdough bread, possibly related to acids produced during sourdough development. Yu et al., (2019) reported that organic acid produced by lactic acid bacteria decreased pH values in bread [27], while Bartkiene et al., (2017) found a strong negative correlation between pH and amylolytic enzyme activity, with activities reported at a high level in *L. plantarum* sourdough [51].

Specific volume and texture profiles of chewiness, hardness, springiness and cohesiveness showed insignificant (*p* ≥ 0.05) differences between common sourdough bread and fermented water sourdough bread. Mohd Roby et al., (2020) reported a similar result, indicating that encapsulated kombucha sourdough bread exhibited comparably specific loaf volume as liquid traditional sourdough bread [22]. The specific volume of our fermented water sourdough bread was 1.2 times higher than encapsulated kombucha sourdough bread [22], suggesting that the fermented water sourdough bread acidified dough had an improved gas-holding gluten capacity [12,52].

#### 3.2.2. Nutritional Quality

Nutritional compositions of common sourdough bread and fermented water sourdough bread are shown in Table 3. Energy, moisture content, protein, total fat, carbohydrate and ash contents insignificantly differed between common sourdough bread and fermented water sourdough bread. However, fermented water sourdough bread showed significantly (*p* < 0.05) lower total sugar content than common sourdough bread, related to higher numbers of *L. plantarum* and *S. cerevisiae* in fermented water sourdough (Figure 1). Most starches are hydrolyzed by lactic acid bacteria during sourdough fermentation, resulting in digestible sugar for yeast consumption, especially glucose, as the primary carbon source for yeast survival and, thus, lower total sugar content [53,54]. Yoon et al., (2003) also suggested that fermentation by *S. cerevisiae* completely removed disaccharides such as maltose, sucrose and turanose, while cellobiose, lactose and melibiose levels were maintained [55]. Similarly, Jin et al., (2019) indicated that total soluble solids and reducing sugar content of mango pulp fermented with *S. cerevisiae* DV10 (single and co-culture) significantly (*p* < 0.05) decreased after 24 h fermentation, while these values remained unchanged in mango pulp fermented by only *L. plantarum* [40]. Total dietary fiber in fermented water sourdough bread was significantly (*p* < 0.05) higher than in common sourdough bread (Table 3) because the insoluble dietary fiber in the fermented water could not be digested by microorganisms [52].

#### 3.2.3. Sensory Evaluation

**Figure 3:**
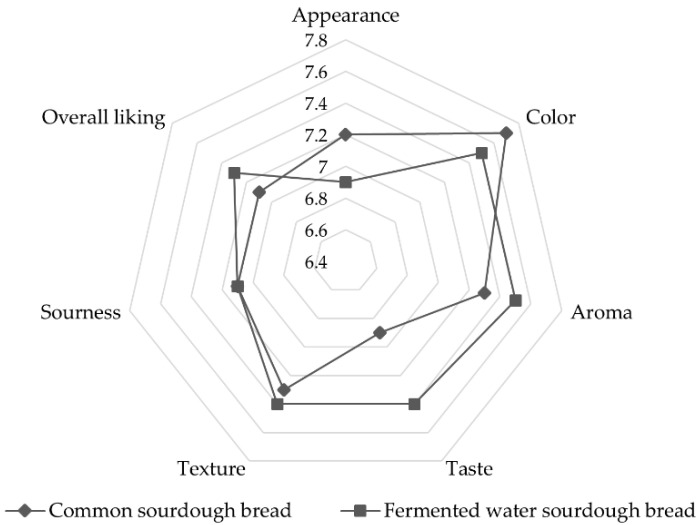
Sensory properties of common sourdough bread and fermented water sourdough bread.

Consumer acceptability is an important factor when developing food products because this influences consumer purchase willingness. A hedonic scale sensory analysis was performed by trained panelists (*n* = 10), with results depicted in Figure 3. Both common and fermented water sourdough bread samples were well accepted by the panelists, with all attribute scores higher than 6 [56,57,58]. The fermented water sourdough bread obtained higher scores for aroma, taste, texture and overall liking than common sourdough bread. This can be due to the fruity and unique aroma of pear and Assam tea, respectively. The advantage of using aromatic raw materials in sourdough bread was reported also in a study by Karimi et al., (2020). It was suggested that sourdough bread containing pacific white shrimp (*Litopenaeus vannamei*) protein hydrolysates and (−)-epigallocatechin gallate had significantly (*p* < 0.05) higher preference scores of taste, flavor, softness, chewiness and overall acceptability than the control bread [11]. Insignificantly different sourness scores between the bread samples were observed although the pH values of fermented water sourdough bread were lower than common sourdough bread (Table 2). It indicated that flavor and acidic taste from fermentation with Asian pears and Assam tea leaves in fermented water sourdough bread did not adversely affect sensory perceptions. Sensory scores on appearance and color of common sourdough bread were higher than fermented sourdough bread due to the mild-yellow color of fermented sourdough bread from the color of fermented water. The overall liking score obtained for fermented sourdough bread was significantly higher than common sourdough bread. The fermented sourdough bread recorded a very satisfactory score of 7.3, considering that it was a new product.

### 3.3. Shelf-Life Stability

#### 3.3.1. Microbiological Quality

Microbial analysis results of common sourdough bread and fermented water sourdough bread under different storage times are shown in Table 4. The initial viable cell counts of total aerobic bacteria, yeast and mold in both common sourdough bread and fermented water sourdough bread on a baking day passed the criteria of the Thai community product standard. This states that total aerobic bacteria must be less than 4 Log CFU/g and less than 2 Log CFU/g for yeast and mold [59]. During storage, aerobic bacteria grew faster than yeast and mold. At day 7 of storage time, total aerobic bacteria were detected at 4.26 Log CFU/g in common sourdough bread, while only 3.38 Log CFU/g was detected in fermented water sourdough bread. This was due to bacteriocin which is produced from *L. plantarum* during fermentation. Behera, Ray and Zdolec (2018) stated that *L. plantarum* produced bacteriocin of high activity and a wide range of antimicrobial activity and their properties could increase shelf life of products [60]. Epigallocatechin gallate (EGCG), a predominant phenolic in Assam tea, also possessed antimicrobial activities [61,62].

Yeast and mold were found at less than 1 Log CFU/g in both common sourdough bread and fermented water sourdough bread over the whole storage period (Table 4). The presence of *L. plantarum* in both mature fermented water sourdough and common sourdough prevented the growth of fungi by producing antifungal substances such as cyclic dipeptides, hydroxy fatty acids or phenyl and substituted phenyl derivates (3-phenyllactic acid, 4-hydroxyphenyl acetic acid and benzoic acid) [63]. Our results showed that fermented water sourdough increased bread shelf-life by 2 days at room temperature (7 days) compared to common sourdough bread (5 days).

#### 3.3.2. Total Phenolic Contents and Antioxidant Activities

TPCs and antioxidant activities detected by ORAC, FRAP and DPPH radical scavenging assays of fermented water sourdough bread were significantly (*p* < 0.05) higher than the common sourdough bread throughout shelf-life storage times (Table 5). The lower pH of fermented water sourdough bread was beneficial for the presence of TPCs and antioxidant activities because polyphenols were auto-oxidized at higher pH [38]. However, TPCs and antioxidant activities of both fermented water sourdough bread and common sourdough bread significantly (*p* < 0.05) declined during storage. The degradation of TPCs and antioxidant activities was due to high temperature, change in pH or oxygen availability [64].

## 4. Conclusions

This study showed that fermented water sourdough passed the criteria of mature sourdough after 4 h, following 18 h retard fermentation, with significantly higher growth of *S. cerevisiae*, optimal pH and improved antioxidant activity compared with common sourdough. The fermented water sourdough bread had 10% less sugar, 12% higher dietary fiber and 2 to 3 times higher total phenolic contents and antioxidant activities detected by ORAC, FRAP and DPPH radical scavenging assays, compared with common sourdough bread. Sensory evaluation determined that the fermented water sourdough bread was preferred in terms of aroma, taste, texture and overall liking when compared with common sourdough bread. The fermented water sourdough bread presented good stability during room temperature (28 ± 2 °C and 75 ± 5% RH) storage for 7 days. At the end of the storage period, adequate amounts of bioactive compounds and good microbial quality suggested that the bread could be safely consumed. The finding demonstrated that fermented water prepared from Asian pears and Assam tea leaves using *L. plantarum* 299v and *S. cerevisiae* TISTR 5059 as starter cultures was successfully applied to develop sourdough and bread with improved qualities. However, the significant improvement found in the current study needs to be validated at the industrial application level.

## Figures and Tables

**Table 1:** Total phenolic contents (TPCs) and antioxidant activities of common sourdough and fermented water sourdough fermented at different time periods (0 and 4 h).

Common Sourdough				
**Time (h)**	**TPCs** **(mg GAE/g DW)**	**Antioxidant Activities**		
		**ORAC Assay** **(μmol TE/g DW)**	**FRAP Assay** **(μmol TE/g DW)**	**DPPH Radical Scavenging Assay** **(μmol TE/100 g DW)**
**0**	0.24 ± 0.02 ^b^	9.88 ± 1.16 ^b^	2.51 ± 0.07 ^b^	0.16 ± 0.01 ^b^
**4**	0.50 ± 0.05 ^a^	17.64 ± 1.22 ^a^	4.20 ± 0.29 ^a^	0.50 ± 0.04 ^a^
**Fermented Water Sourdough**				
**Time (h)**	**TPCs** **(mg GAE/g DW)**	**Antioxidant Activities**		
		**ORAC assay** **(μmol TE/g DW)**	**FRAP assay** **(μmol TE/g DW)**	**DPPH radical scavenging assay** **(μmol TE/100 g DW)**
**0**	1.76 ± 0.03 ^b,^*	91.36 ± 4.31 ^b,^*	19.40 ± 0.27 ^a,^*	0.81 ± 0.08 ^b,^*
**4**	2.52 ± 0.24 ^a,^*	121.55 ± 6.93 ^a,^*	17.74 ± 0.74 ^b,^*	1.19 ± 0.08 ^a,^*

All data are shown as the mean ± standard deviation (SD) of triplicate determinations (*n* = 3). Different lowercase letters denote significantly different TPCs or antioxidant activities at *p* < 0.05 of the same type of sourdough fermented at different time periods, while * denotes significant differences at *p* < 0.05 between common sourdough and fermented water sourdough fermented at the same time period using Student’s unpaired *t*-test. ORAC: oxygen radical absorbance capacity; FRAP: ferric ion reducing antioxidant power; DPPH: 2,2-diphenyl-1-picrylhydrazyl; GAE: gallic acid equivalent; TE: Trolox equivalent; DW: dry weight.

**Table 2:** pH, specific volume and texture profile of common sourdough bread and fermented water sourdough bread.

Properties	Common Sourdough Bread	Fermented Water Sourdough Bread	Significance
pH	4.12 ± 0.01 *	4.02 ± 0.01	t = 22.627; sig. = 0.000
Specific volume (mL/g)	5.22 ± 0.05	5.28 ± 0.11	t = −0.873; sig. = 0.432
Water activity	0.82 ± 0.00	0.82 ± 0.00	t = 0.000; sig. = 1.000
Texture profile			
Hardness (g)	268.06 ± 17.81	248.54 ± 30.49	t = 0.958; sig. = 0.392
Chewiness	205.69 ± 1.78	192.62 ± 17.87	t = 1.261; sig. = 0.332
Springiness (cm)	0.92 ± 0.03	0.92 ± 0.02	t = −0.085; sig. = 0.936
Cohesiveness (g)	0.84 ± 0.02	0.85 ± 0.02	t = −0.387; sig. = 0.719

All data are shown as the mean ± standard deviation (SD) of triplicate determinations (*n* = 3). The * denotes significantly different values at *p* < 0.05 of the same property in common sourdough bread and fermented water sourdough bread using Student’s unpaired *t*-test.

**Table 3:** Nutritional values of common sourdough bread and fermented water sourdough bread.

Nutrients (per 100 g FW)	Common Sourdough Bread	Fermented Water Sourdough Bread	Significance
Energy (kcal)	271.24 ± 0.66	273.75 ± 1.07	t = −2.831; sig. = 0.105
Moisture (%)	33.76 ± 0.28	33.16 ± 0.45	t = 1.620; sig. = 0.247
Protein (N × 6.25) (g)	9.73 ± 0.07	9.79 ± 0.10	t = −0.697; sig. = 0.558
Total fat (g)	2.80 ± 0.11	2.83 ± 0.15	t = −0.232; sig. = 0.838
Total carbohydrate (g)	51.78 ± 0.33	52.28 ± 0.70	t = -0.912; sig. = 0.458
Total dietary fiber (g)	1.54 ± 0.00	1.73 ± 0.04 *	t = −6.333; sig. = 0.024
Total sugar (g)	8.94 ± 0.07 *	8.09 ± 0.01	t = 16.866; sig. = 0.003
Ash (g)	1.93 ± 0.02	1.94 ± 0.01	t = −0.632; sig. = 0.592

All data are shown as the mean ± standard deviation (SD) of triplicate determinations (*n* = 3). The * denotes significantly different contents at *p* < 0.05 of the same nutritional composition in common sourdough bread and fermented water sourdough bread using Student’s unpaired *t*-test.

**Table 4:** Total aerobic bacteria and yeast and mold of common sourdough bread and fermented water sourdough bread over storage duration.

Storage Days	Common Sourdough Bread		Fermented Water Sourdough Bread	
	Total Aerobic Bacteria (Log CFU/g)	Yeast and Mold (Log CFU/g)	Total Aerobic Bacteria (Log CFU/g)	Yeast and Mold (Log CFU/g)
0	0.00 ± 0.00 ^d^	Less than 1	2.57 ± 0.01 ^b,^*	Less than 1
1	0.00 ± 0.00 ^d^	Less than 1	2.37 ± 0.13 ^bc,^*	Less than 1
3	2.98 ± 0.03 ^c,^*	Less than 1	2.30 ± 0.14 ^c^	Less than 1
5	3.23 ± 0.01 ^b^	Less than 1	3.21 ± 0.02 ^a^	Less than 1
7	4.26 ± 0.06 ^a,^*	Less than 1	3.38 ± 0.02 ^a^	Less than 1

All data are shown as the mean ± standard deviation (SD) of duplicate determinations. Different lowercase letters denote significantly different viable cell count of microorganisms at *p* < 0.05 of the same type of bread under different storage times using one-way ANOVA, followed by Duncan’s multiple comparison test, while * denotes significantly different viable cell count of microorganisms at *p* < 0.05 between common sourdough bread and fermented water sourdough bread at the same storage time using Student’s unpaired *t*-test.

**Table 5:** Total phenolic contents (TPCs) and antioxidant activities detected by ORAC, FRAP, and DPPH radical scavenging assays of bread prepared by common sourdough and fermented water sourdough over storage duration.

Common Sourdough Bread				
Storage Days	TPCs (mg GAE/g DW)	Antioxidant Activities		
		ORAC Assay (μmol TE/g DW)	FRAP Assay (μmol TE/g DW)	DPPH Radical Scavenging Assay (μmol TE/100 g DW)
**0**	0.28 ± 0.02 ^a^	6.04 ± 0.60 ^ab^	0.98 ± 0.07 ^a^	0.16 ± 0.01 ^a^
**1**	0.27 ± 0.03 ^a^	6.44 ± 0.60 ^a^	0.83 ± 0.03 ^b^	0.12 ± 0.01 ^c^
**3**	0.24 ± 0.01 ^b^	6.10 ± 0.57 ^ab^	0.74 ± 0.04 ^c^	0.13 ± 0.01 ^b^
**5**	0.25 ± 0.01 ^b^	5.88 ± 0.29 ^ab^	0.70 ± 0.02 ^d^	0.14 ± 0.01 ^b^
**7**	0.23 ± 0.02 ^b^	5.79 ± 0.33 ^b^	0.75 ± 0.03 ^c^	0.12 ± 0.01 ^c^
**Fermented Water Sourdough Bread**				
**Storage Days**	**TPCs** **(mg GAE/g DW)**	**Antioxidant Activities**		
		**ORAC assay** **(μmol TE/g DW)**	**FRAP assay** **(μmol TE/g DW)**	**DPPH radical scavenging assay** **(μmol TE/100 g DW)**
**0**	0.68 ± 0.02 ^a^*	14.89 ± 1.44 ^c,^*	3.12 ± 0.22 ^a,^*	0.33 ± 0.03 ^a,^*
**1**	0.66 ± 0.03 ^ab,^*	26.73 ± 0.65 ^a,^*	3.08 ± 0.29 ^a,^*	0.29 ± 0.01 ^b,^*
**3**	0.64 ± 0.03 ^b^*	26.54 ± 2.63 ^a,^*	2.77 ± 0.18 ^bc,^*	0.29 ± 0.03 ^b,^*
**5**	0.61 ± 0.02 ^c,^*	23.45 ± 0.93 ^b,^*	2.66 ± 0.08 ^c,^*	0.29 ± 0.02 ^b,^*
**7**	0.57 ± 0.05 ^d,^*	15.26 ± 1.47 ^c,^*	2.90 ± 0.11 ^b,^*	0.27 ± 0.02 ^c,^*

All data are shown as the mean ± standard deviation (SD) of triplicate determinations (*n* = 3). Different lowercase letters denote significantly different TPCs or antioxidant activities at *p* < 0.05 of the same type of sourdough bread stored at different time periods, while * denotes significant differences at *p* < 0.05 between common sourdough bread and fermented water sourdough bread stored at the same time period using Student’s unpaired *t*-test. ORAC: oxygen radical absorbance capacity; FRAP: ferric ion reducing antioxidant power; DPPH: 2,2-diphenyl-1-picrylhydrazyl; GAE: gallic acid equivalent; TE: Trolox equivalent; DW: dry weight.

## Data Availability

Data are contained within this article.

## Supplementary Materials

The following supporting information can be downloaded at: https://www.mdpi.com/article/10.3390/foods11142071/s1, Supplementary Table S1. Growth of *L. plantarum* and *S. cerevisiae* in common sourdough and fermented water sourdough throughout fermentation; Supplementary Table S2. A trend in pH and height of common sourdough and fermented water sourdough over fermentation time periods.
