# Supplementary material for: Improvement of Sourdough and Bread Qualities by Fermented Water of Asian Pears and Assam Tea Leaves with Co-Cultures of Lactiplantibacillus plantarum and Saccharomyces cerevisiae
Source: Foods. 2022 Jul 12;11(14):2071. doi: 10.3390/foods11142071 (PMC9318377; doi:10.3390/foods11142071)
Supplement: Supplementary file 1 [file foods-11-02071-s001.zip › foods-1801138-supplementary.pdf]

foods

Supplementary materials

The Improvement of Sourdough and Bread Qualities by Fermented Water from Asian Pears and Assam Tea Leaves with Co-cultures of *Lactiplantibacillus plantarum* and *Saccharomyces cerevisiae*

Ramita Supasil, Uthaiwan Suttisansanee, Chalat Santivarangkna, Nattapol Tangsuphoom, Chanakan Khemthong, Chaowanee Chupeerach and Nattira On-nom *

Food and Nutrition Academic and Research Cluster, Institute of Nutrition, Mahidol University, Salaya, Phuttamonthon, Nakhon Pathom 73170, Thailand; ramita.sup1@gmail.com (R.S.); uthaiwan.sut@mahidol.ac.th (U.S.); chalat.san@mahidol.ac.th (C.S.); mailto:nattapol.tng@mahidol.ac.th (N.T.); chanakan.khe@mahidol.ac.th (C.K.); chaowanee.chu@mahidol.ac.th (C.C.)

* Correspondence: nattira.onn@mahidol.ac.th (N.O.); Tel.: +66-(0)-2800-2380 (ext.426)

Table S1. Growth of *L. plantarum* and *S. cerevisiae* in common sourdough and fermented water sourdough throughout fermentation.

Fermentation time (h)	*L. plantarum*		*S. cerevisiae*	
	Common sourdough	Fermented water sourdough	Common sourdough	Fermented water sourdough
0	7.28 ± 0.05 ^{b}	7.27 ± 0.10 ^{c}	5.75 ± 0.02 ^{c}	5.72 ± 0.04 ^{c}
1	7.22 ± 0.04 ^{b}	7.83 ± 0.10 ^{b,*}	5.95 ± 0.06 ^{a}	6.11 ± 0.07 ^{b,*}
2	7.39 ± 0.08 ^{b}	7.87 ± 0.08 ^{b,*}	5.81 ± 0.06 ^{bc}	6.15 ± 0.02 ^{b,*}
3	7.39 ± 0.14 ^{b}	7.82 ± 0.10 ^{b,*}	5.84 ± 0.02 ^{b}	6.15 ± 0.12 ^{b,*}
4	7.94 ± 0.27 ^{a}	8.14 ± 0.07 ^{a}	6.00 ± 0.04 ^{a}	6.36 ± 0.03 ^{a,*}

Results were expressed as mean values of triplicate determinations (n =3). Different uppercase and lowercase letters denote significantly different viable cell count of microorganisms in common sourdough and fermented water sourdough, respectively at different fermentation times using one-way ANOVA, followed by Duncan's multiple comparison test at $p <$ 0.05, while * denotes significantly different viable cell count of microorganisms between common sourdough and fermented water sourdough detected at the same fermentation time using Student's unpair t-test at $p < 0.05$.

Table S2. A trend in pH and height of common sourdough and fermented water sourdough over fermentation time periods.

Fermentation time (h)	pH		Height (inch)	
	Common sourdough	Fermented water sourdough	Common sourdough	Fermented water sourdough
0	5.98 ± 0.01 ^{a,*}	5.51 ± 0.02 ^{a}	1.00 ± 0.00 ^{d}	1.00 ± 0.00 ^{d}
1	5.36 ± 0.15 ^{b,*}	4.48 ± 0.05 ^{b}	1.25 ± 0.25 ^{cd}	1.50 ± 0.87 ^{cd}
2	4.94 ± 0.02 ^{c,*}	4.46 ± 0.04 ^{b}	1.58 ± 0.38 ^{c}	2.17 ± 0.58 ^{bc}
3	4.80 ± 0.05 ^{d,*}	4.21 ± 0.03 ^{c}	2.00 ± 0.50 ^{b}	2.83 ± 0.76 ^{ab}
4	4.45 ± 0.02 ^{e,*}	4.14 ± 0.03 ^{d}	2.50 ± 0.50 ^{a}	3.33 ± 0.76 ^{a}

Results were expressed as mean values of triplicate determinations (n = 3). Different uppercase and lowercase letters denote significantly different values (pH or height) at $p < 0.05$ in common sourdough and fermented water sourdough, respectively, fermented at different time periods using one-way ANOVA, followed by Duncan's multiple comparison test, while * denotes significantly different values (pH or height) at $p < 0.05$ between common sourdough and fermented water sourdough fermented at the same time period using Student's unpaired t-test.
